# Soft Tissue Extramedullary Plasmacytoma

**DOI:** 10.1155/2010/307902

**Published:** 2010-03-03

**Authors:** Fernando Ruiz Santiago, Manuel Tello Moreno, Aurelio Martín Castro, Luis Guzmán Álvarez, Pedro Navarrete González

**Affiliations:** ^1^Department of Radiology, Hospital of Traumatology (Ciudad Sanitaria Virgen de las Nieves), Carretera de Jaen, SN, 18013 Granada, Spain; ^2^Department of Pathology, Hospital of Traumatology (Ciudad Sanitaria Virgen de las Nieves), Carretera de Jaen, SN, 18013 Granada, Spain

## Abstract

We present the uncommon case of a subcutaneous fascia-based extramedullary plasmacytoma in the leg, which was confirmed by the pathology report and followed up until its remission. We report the differential diagnosis with other more common soft tissue masses. Imaging findings are nonspecific but are important to determine the tumour extension and to plan the biopsy.

## 1. Introduction

Extramedullary plasmacytoma (EMP) accounts for 3% of myeloma cases and is usually localized in submucosal lymphoid tissue of nasopharyngeal and paranasal sinuses [1]. Soft tissue localization elsewhere is extremely uncommon, and other more common types of soft tissue mass are initially suspected. We present a case of soft tissue plasmacytoma in the leg, confirmed by the pathology report and followed up until its remission. 

## 2. Case Report

**Figure 1:**
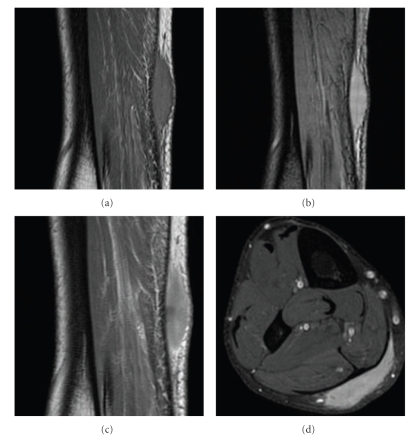
Sagittal T1 (a), T2* (b) weighted images depict a fusiform mass over the Achilles tendon. Postcontrast sagittal T1 (c) and axial fat-saturated T1-weighted images demonstrate a moderate and homogeneous enhancement.

A 52-year-old male was referred to the orthopaedic surgeon for evaluation of a painless bulge in his calf detected 1 year earlier. An MRI was performed in a Signa 1.5 T GE, obtaining axial and sagittal T1- (FSE, TE: 25; TR: 540, ETL: 4) and T2*- (GE, TE: 15; TR: 620 ms, flip angle: 20) weighted images. A homogeneous, oblong and well-defined mass was detected superficial to the Achilles tendon. It was isointense on T1-weighted images and hyperintense on T2*-weighted images and showed homogeneous moderate enhancement after intravenous gadolinium injection (Figure 1).

**Figure 2:**
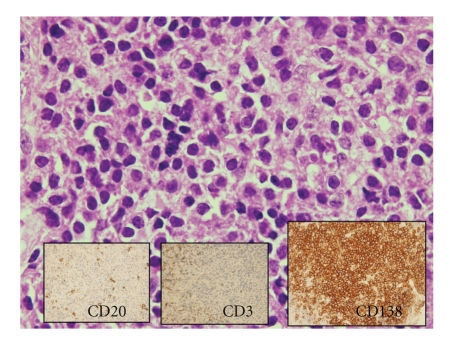
Neoplastic proliferation of differentiated plasma cells. Immunochemistry was positive for CD138 (plasma cell marker) and negative for CD20 (accompanying B-lymphocytes) and CD3 (accompanying T-lymphocytes).

A Tru-Cut biopsy was performed, and the pathology report described a neoplastic proliferation of differentiated plasma cells. Immunochemistry studies showed cells to be positive to CD138 (plasma cell marker) and negative to CD20 (accompanying B-lymphocytes) and CD3 (accompanying T-lymphocytes). These findings supported the diagnosis of plasmacytoma (Figure 2). The presence of any other abnormality was ruled out by subsequent haematological study (bone marrow biopsy, complete blood count and electrophoresis) and PET-CT examination.

The patient was treated by radiotherapy. A very small residual flat thickening of soft tissues, which shows no activity on PET-CT, remains unchanged after four years of followup. The patient is considered to be in complete remission.

## 3. Discussion

Myeloma usually appears as a generalized disease (multiple myeloma). In a small proportion of patients (<5%), it can present as solitary bone plasmacytoma (SBP) or a solitary soft tissue mass, extramedullary plasmacytoma (EMP) [2].

Primary plasmacytoma, whether osseous or nonosseous, is distinguished from multiple myeloma by absence of hypercalcaemia, renal insufficiency and anaemia, normal skeletal survey, absence of bone marrow plasmacytosis, and serum or urinary paraprotein <2 g/dL [3].

The diagnosis of EMP requires demonstration of a monoclonal plasma cell infiltrate with no evidence of myeloma at any other site. Therefore, patients with presumed solitary plasmacytoma of bone or soft tissue (extramedullary) plasmacytoma should undergo a radiological bone survey and MR imaging to ensure that only one lesion is present. Imaging studies should focus on the early detection of additional or recurrent lesions and the presence of regional lymphadenopathy, which will influence the clinical management.

Solitary plamocytomas can arise at any site, but almost 90% develop in the head and neck area and especially in the upper respiratory tract (nose, paranasal sinuses, nasopharynx and tonsils). Infrequent sites of involvement include the gastrointestinal tract, liver, spleen, pancreas, lungs, thyroid, breast, testis or skin [4, 5]. Solitary plasmacytoma presenting as a soft tissue mass is therefore a rare event and not usually included in the differential diagnosis of other more common soft tissue masses, such as sarcomas, fibrous tumours, haemangiomas, neurofibromas or lymphomas.

Malignant fibrous histiocytoma is the most frequent mesenchymal malignancy and accounts for around 24% of all soft-tissue sarcomas, with 7–10% of cases confined to the subcutaneous tissue, where it may or not extend to the fascia. Subcutaneous lesions tend to be smaller, implying a better prognosis. MR imaging of soft-tissue malignant fibrous histiocytomas reveals lesions with intermediate to low signal intensity on T1-weighted images and inhomogeneous high signal intensity on T2-weighted images. Inhomogeneity may be due to calcification, myxoid degeneration with cystic cavities or necrosis. After gadolinium administration, irregular enhancement is seen in the viable tumour [6].

Synovial sarcomas account for around 10% of all soft-tissue sarcomas. The MR signal is usually complex, due to the presence of fluid levels, haemorrhage and internal septation. Neoplasms with a diameter <5 cm may have a more benign appearance, with well-circumscribed margins and homogeneous high signal intensity on T2-weighted images [6].

Among fibrous tumours, nodular fasciitis can present as a localized and circumscribed subcutaneous fascia-based mass. The histologic diversity of nodular fasciitis likely accounts for the variable MR appearance of the lesions. In hypercellular lesions, they can appear isointense to slightly hyperintense relative to skeletal muscle on T1-weighted images and hyperintense to adipose tissue on T2-weighted images. Highly collagenous lesions show a hypointense signal on all MR images. Contrast enhancement is typically diffuse and homogeneous, except in cases of myxoid degeneration leading to cystic degeneration [7].

Soft-tissue haemangiomas can arise at various anatomic sites, including striated muscle, skin, subcutaneous tissue and synovial tissue, depending on the histologic subtype. If sufficiently large, they can manifest as a smooth, palpable soft-tissue mass. MR imaging reflects the mix of tissues present in the haemangioma. It usually shows heterogeneity, due to the variable amount of fat, arteries and veins, which can present a serpentine pattern. Nevertheless, small lesions with no fat content can have a homogeneous signal and well-defined border [8].

Subcutaneous neurofibromas are usually associated with neurofibromatosis-1. An entering and exiting nerve cannot usually be identified in superficial lesions. They generally present as a fusiform-shaped mass with a signal that is isointense to skeletal muscle on T1-weighted MR images and hyperintense to skeletal muscle on T2-weighted images, with variable degrees of inhomogeneity and enhancement [8].

Musculoskeletal involvement by lymphoma can occur in disseminated disease or as an isolated manifestation (primary lymphoma of bone, muscle, cutaneous or subcutaneous tissue). The skin is the second most common site of extranodal involvement of Non-Hodgkin Lymphoma after the gastrointestinal tract. Imaging features of cutaneous lymphoma are nonspecific and include soft tissue thickening, infiltration or a mass [9].

Most of the above diagnoses have to be considered before soft tissue EMP is diagnosed. Although EMP findings are not specific, imaging can be used to delineate the extent of the mass, plan a biopsy and rule out other lesions. In MRI studies, it has been described as a mass that is isointense or slightly hyperintense on T1-weighted images and isointense to high hyperintense on T2-weighted images, with homogeneous-to-heterogeneous enhancement on post-contrast T1-weighted images [1, 10]. Its appearance is influenced by its size, with small lesions visualized as well defined and homogeneous, while larger lesions tend to be invasive and heterogeneous due to the development of areas of necrosis.

The prognosis is significantly better for EMP patients than for SBP patients, with two-thirds surviving >10 years [11], and currently available radiotherapy techniques have minimized the likelihood of local recurrence [12]. The risk of distant metastasis or conversion to multiple myeloma, is <30%. Distant metastases tend to appear within 2-3 years of the initial diagnosis, and a close follow-up is warranted during this period [13].

In summary, we present a rare case of subcutaneous fascia-based EMP, reporting the differential diagnosis with other more common soft tissues mass. Imaging findings are nonspecific but invaluable for studying the extent of the tumour and planning the biopsy.
